# Nanomaterials for Smart and Sustainable Food Packaging: Nano-Sensing Mechanisms, and Regulatory Perspectives

**DOI:** 10.3390/foods14152657

**Published:** 2025-07-29

**Authors:** Arjun Muthu, Duyen H. H. Nguyen, Chaima Neji, Gréta Törős, Aya Ferroudj, Reina Atieh, József Prokisch, Hassan El-Ramady, Áron Béni

**Affiliations:** 1 Doctoral School of Nutrition and Food Science, University of Debrecen, 4032 Debrecen, Hungary; arjun.muthu@agr.unideb.hu (A.M.); nguyen.huu.huong.duyen@agr.unideb.hu (D.H.H.N.); atieh.reina@mailbox.unideb.hu (R.A.); 2 Institute of Agricultural Chemistry and Soil Science, Faculty of Agricultural and Food Sciences and Environmental Management, University of Debrecen, 138 Böszörményi Street, 4032 Debrecen, Hungary; 3 Institute of Animal Science, Faculty of Agricultural and Food Sciences and Environmental Management, Biotechnology and Nature Conservation, University of Debrecen, 138 Böszörményi Street, 4032 Debrecen, Hungary; toros.greta@agr.unideb.hu (G.T.); ferroudj.aya@agr.unideb.hu (A.F.); jprokisch@agr.unideb.hu (J.P.); hassan.elramady@agr.kfs.edu.eg (H.E.-R.); 4 Institute of Life Sciences, Vietnam Academy of Science and Technology, 9/621 Vo Nguyen Giap Street, Linh Trung Ward, Thu Duc City 721400, Ho Chi Minh, Vietnam; 5 Institute of Nutrition, Doctoral School of Nutrition and Food Science, Faculty of Agricultural and Food Sciences and Environmental Management, University of Debrecen, 138 Böszörményi Street, 4032 Debrecen, Hungary; neji.chaima@agr.unideb.hu; 6 Doctoral School of Animal Husbandry, Faculty of Agricultural and Food Sciences and Environmental Management, University of Debrecen, Böszörményi Street 138, 4032 Debrecen, Hungary; 7 Soil and Water Department, Faculty of Agriculture, Kafrelsheikh University, Kafr El-Sheikh 33516, Egypt

**Keywords:** active and intelligent packaging, food safety, food system, circular economy

## Abstract

The global food industry is facing growing pressure to enhance food safety, extend shelf life, minimize waste, and adopt environmentally sustainable packaging solution. Nanotechnology offers innovative ways to meet these demands by enabling the creation of smart and sustainable food packaging systems. Due to their unique properties, nanomaterials can significantly enhance the functional performance of packaging by boosting mechanical strength, barrier efficiency, antimicrobial activity, and responsiveness to environmental stimuli. This review provides a comprehensive overview of nanomaterials used as smart and sustainable food packaging, focusing on their role in active and intelligent packaging systems. By integrating nanomaterials like metal and metal oxide nanoparticles, carbon-based nanostructures, and nano-biopolymers, packaging can now perform real-time sensing, spoilage detection, and traceability. These systems improve food quality management and supply chain transparency while supporting global sustainability goals. The review also discusses potential risks related to nanomaterials’ migration, environmental impact, and consumer safety, as well as the current regulatory landscape and limitations in industrial scalability. Emphasis is placed on the importance of standardized safety assessments and eco-friendly design to support responsible innovation. Overall, nano-enabled smart packaging represents a promising strategy for advancing food safety and sustainability. Future developments will require collaboration across disciplines and robust regulatory frameworks to ensure the safe and practical application of nanotechnology in food systems.

## 1. Introduction

The global food industry is facing enormous challenges concerning food safety, quality preservation, sustainability, and supply chain management transparency [1]. Traditional food packaging, primarily composed of petroleum-based plastics, offers basic protection but lacks biodegradability, contributes significantly to environmental pollution, and falls short in actively preserving food quality [2]. These limitations necessitate the development of advanced packaging solutions. Nanoparticles (NPs) are typically defined as materials with at least one dimension between 1 and 100 nm. They can exhibit unique properties, including a high surface area-to-volume ratio, quantum effects, and adjustable optical, electrical, and mechanical properties [3,4]. Materials smaller than 10 nm are often called quantum dots or nanodots (i.e., quasi-zero-dimensional NPs exhibiting quantum effects), while those larger than 100 nm exhibit bulk-like behavior with diminished surface-to-volume effects and reduced quantum properties [5,6,7]. Incorporating nanomaterials into food packaging offers excellent opportunities to improve functionality and sustainability [8]. These materials can increase mechanical strength, barrier performance, antimicrobial activity, and responsiveness to environmental changes, thereby extending shelf life, reducing spoilage, and improving food safety [9,10]. Using them in biodegradable matrices also supports the growing global demand for alternatives to petroleum-based conventional plastics, aligning with the circular economy and sustainability goals [11].

Alongside advancements in nanotechnology, the rise of smart materials and devices has expanded the potential of modern packaging systems [12]. Smart materials can respond to environmental changes, such as temperature, pH, gas composition, or humidity, through controlled and predictable changes in their optical, thermal, electrical, or magnetic properties [13]. When combined with nanotechnology, this gives rise to smart nanomaterials, a class of engineered nano systems that can sense, respond to, and communicate changes within packaged food environments [10]. This interconnection has led to the development of smart and sustainable food packaging, where nanomaterials not only serve as passive reinforcements but also enable active and intelligent functionalities such as spoilage detection, real-time freshness monitoring, and traceability through embedded nanosensors [14].

This review provides a comprehensive overview of the classification, sensing mechanisms, and functional applications of nanomaterials in smart food packaging. The review highlights the role of different types of nanomaterials, such as metals, metal oxides, carbon-based structures, and biopolymeric NPs, in improving food safety, quality, and sustainability. The review also addresses regulatory challenges, toxicity concerns, and knowledge gaps regarding long-term behavior and consumer safety. Finally, it discusses future research directions and outlines strategies to advance safe, scalable, and sustainable nano-enabled packaging solutions suitable for industrial adoption.

## 2. Classification of Smart Food Packaging Systems

**Figure 1:**
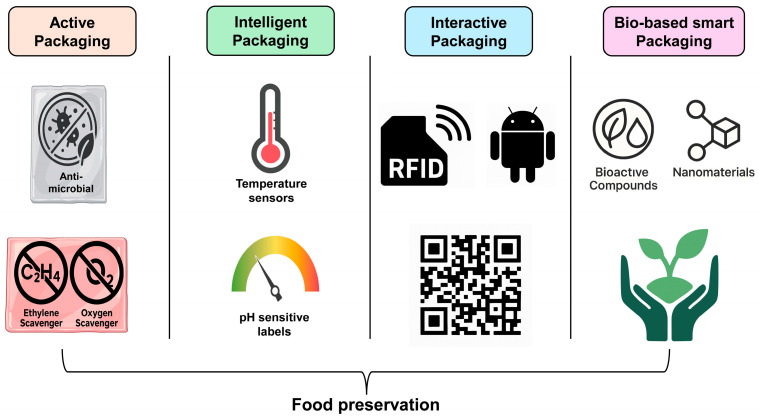
Types of smart food packaging and their key features for food preservation.

Smart food packaging is defined as a packaging system that monitors and evaluates the condition of a food product or its environment [15,16]. Depending on the functionality, smart food packaging can be classified into active, intelligent, and interactive packaging systems. Bio-based smart packaging classification refers to systems made from renewable, biodegradable materials and represents a material-oriented classification that is relevant to sustainability goals. Smart packaging is designed to monitor/track the product, sense the internal or external environment of the package, and communicate with the consumer [17,18]. These monitoring technologies/devices include biosensors, electrochemical sensors, gas sensors, and color indicators, which are illustrated in Figure 1. Various types of smart packaging devices are available in the market, which can be further classified as smart labels or indicators, smart closures, smart bags, and smart trays [16,19].

### 2.1. Active Packaging

Active packaging refers to packaging systems designed to interact with the food or its environment to extend shelf life, maintain quality, and improve safety [10,20]. Unlike passive barriers, active systems incorporate functional agents, such as antimicrobials, oxygen scavengers, or moisture regulators, directly into packaging materials to modulate the internal atmosphere and inhibit spoilage [21]. One approach involves the use of engineered porous materials, such as activated carbon or nano-porous silicates, to adsorb gases (e.g., oxygen, ethylene) and reduce internal emission levels [22]. These gas-regulating systems are particularly effective for fresh produce and high-respiration foods. Another strategy involves biomimetic and multifunctional films, often fabricated from biodegradable polymers and bioactive compounds. These films offer both antimicrobial and barrier properties while aligning with sustainability goals [23]. Natural plant extracts such as clove oil, cinnamon oil, rosemary oil, and oregano oil have been widely studied for their antimicrobial and antioxidant properties. When integrated into edible coatings or films, they help delay microbial growth, slow down enzymatic browning, and extend the shelf life of perishable items like fruits and vegetables [24]. Additionally, nano-metal and metal oxide particles, such as copper oxide (CuO), exhibit potent antimicrobial action by releasing metal ions in a controlled manner. Studies have shown that CuO-based active films significantly reduce total viable bacterial counts, Gram-negative pathogens, algae, and spoilage fungi [25]. Other active agents include powdered natural additives (e.g., essential oil microcapsules), which allow the controlled release of antimicrobials into the food environment, and oxygen-scavenging NPs embedded in polymer matrices. These reduce oxidative degradation and prevent undesirable physicochemical changes such as lipid rancidity, nutrient loss, or color fading [26]. Nano-enabled materials also support the design of UV-resistant, biodegradable coatings, such as henna leaf gum-coated food-grade paper, which serve both protective and sustainability functions. Recent innovations focus on multifunctional nano-packaging materials capable of delivering active compounds like zerumbone or turmeric oil, which help inhibit oxidation and suppress Maillard reactions in heat-processed foods [27,28]. Nanomaterials such as nano-Ag, ZnO, and CuO are frequently integrated into active packaging films for their antimicrobial action. These NPs inhibit microbial proliferation by disrupting cell membranes or generating reactive oxygen species [10]. Others, like nano-clays, are used as oxygen scavengers, delaying oxidation-related spoilage [26]. Together, these developments mark a shift toward high-performance packaging systems that integrate biological activity, barrier enhancement, and environmental safety. In summary, active packaging plays a pivotal role in modern food preservation. It offers real-time protection against spoilage factors while minimizing the environmental impact associated with traditional plastic-based systems. Incorporating nanomaterials can significantly enhance the functionality of active packaging, establishing it as a key enabler of smart and sustainable food packaging systems.

### 2.2. Intelligent Packaging

Intelligent packaging refers to systems that monitor, detect, and communicate real-time information about the condition, quality, or history of food products throughout the supply chain [29]. These systems typically integrate indicators, sensors, and radio-frequency identification (RFID) tags to provide feedback on variables such as temperature, pH, gas composition, and microbial activity [30]. In contrast to passive systems, intelligent packaging enables dynamic interactions with the product and its environment, enhancing decision-making and consumer trust.

An emerging idea is attribute-based sensing, which directly monitors intrinsic food quality parameters like pH, color, or texture through embedded sensors or responsive materials. Unlike traditional packaging that depends on external tags or indicators, this method allows for real-time assessment of food freshness and spoilage [31,32,33]. For instance, pH-sensitive films incorporating red cabbage anthocyanins exhibit visible color changes as spoilage progresses, providing real-time and non-invasive freshness monitoring for meat and seafood products [34]. These biosensors are positioned near the food surface and can function as direct spoilage indicators, reducing the risks of contamination and degradation often associated with external tags or chemical indicators [35]. Beyond freshness indicators, intelligent systems enable additional functionalities such as traceability, anti-counterfeiting, and modified atmosphere packaging (MAP) monitoring [36]. For example, RFID-enabled smart packaging is widely used in cold chain logistics to track temperature history and location from farm to consumer, thereby reducing spoilage and improving transparency [37,38]. The integration of digital readout devices, including smartphone-compatible sensors, further enhances user interaction and accessibility, broadening the scope of intelligent packaging from industry to end-users [39]. Recent advances also explore the synergy between intelligent packaging and biodegradable nanomaterials. Natural polymer-based semi-permeable films, such as those made from chitosan, gelatin, or alginate, can be reinforced with NPs or essential oils to provide both sensory and antimicrobial functionality [40,41]. Metal oxide NPs (e.g., TiO_2_, ZnO) are incorporated into sensors for detecting volatile organic compounds (VOCs) associated with spoilage. Quantum dots and carbon dots also enable fluorescence-based indicators that signal freshness or contamination via color change [42]. Nanomaterial-enhanced sensors, including carbon nanotubes and metal oxide nanowires, are being developed for integration into smart labels, offering high sensitivity and selectivity for gases like ammonia and ethylene. However, the widespread application of nano-enabled intelligent packaging raises concerns related to nanotoxicity, material compatibility, and environmental persistence. Robust risk assessments, including lifecycle analysis and migration studies, are needed to ensure consumer safety and regulatory compliance [43,44,45]. In summary, intelligent packaging transforms conventional food packaging into responsive platforms capable of monitoring, signaling, and tracing food quality in real time. By integrating nanomaterials with biodegradable matrices and digital tools, these systems represent a critical advancement toward sustainable, traceable, and consumer-friendly food packaging solutions.

### 2.3. Interactive Packaging

In today’s consumer-centric food market, there is increasing demand for high-quality, safe products, coupled with expectations for price transparency and ethical sourcing [46]. To meet these expectations, documenting the entire supply chain from agricultural inputs to final retail is essential [47]. This growing emphasis on traceability has accelerated the integration of interactive packaging technologies, which embed digital tools into physical packaging to enhance product transparency, safety, and consumer engagement [48]. Technologies such as Near Field Communication (NFC), QR codes, and smartphone-based scanners allow users to access detailed information about a product’s origin, storage history, expiration status, and even usage suggestions in real time. These platforms also facilitate personalized engagement, including recipe recommendations, sustainability storytelling, and waste-reduction prompts, all of which contribute to consumer trust and food quality assurance [49,50]. Applications of interactive packaging generally fall into three categories:

• Quality assurance and safety: These systems ensure product authenticity, prevent tampering, and monitor packaging integrity. Nanomaterials can enhance this functionality by improving barrier performance and enabling embedded sensing elements [51,52].
• Supply chain monitoring and transparency: Nano-enabled sensors and barriers help track storage and transport conditions, maintaining food quality across complex logistics networks [37].
• Marketing and consumer engagement: Tools such as augmented reality (AR), QR codes, and connected storytelling platforms are used to create immersive brand interactions and highlight environmental or nutritional credentials [39].

Recent advances incorporating nanomaterials into interactive packaging have extended its capabilities. For instance, carbon nanotubes (CNTs) and graphene-based nanosensors embedded in smart labels can detect gases such as ethylene or ammonia, enabling freshness tracking through smartphone interfaces [53]. Nanocomposites containing carbon nanotubes or graphene are used in printed sensors and RFID-embedded labels for real-time data collection and transmission. These materials improve the electrical conductivity and durability of smart labels used for traceability and consumer engagement [54]. Simultaneously, packaging sustainability is being reinforced through servitization strategies, business models that shift from selling physical products to delivering services, such as refillable containers, packaging reuse programs, or subscription-based systems designed for reduced material consumption and enhanced customer value. It emphasizes reuse, repair, and modularity [55]. Innovations include refillable containers, reusable bottles, and modular packaging components designed for easy disassembly. Circular business models, such as subscription-based delivery systems and packaging buy-back programs, are supported by biodegradable nanocomposites, which allow repeated use and end-of-life composting, and by intelligent refill tracking systems that monitor usage and encourage reuse through digital incentives or automated refills, thereby minimizing waste and promoting resource efficiency [56,57]. Collectively, these innovations signal a shift toward more transparent, user-responsive, and sustainable packaging ecosystems, integrating digital functionality with materials science. Interactive packaging, particularly when reinforced by nanotechnology, represents a promising avenue for meeting evolving consumer needs and aligning with global sustainability priorities [58].

### 2.4. Bio-Based Smart Packaging

The global demand for bio-based packaging is steadily rising as an environmentally friendly alternative to conventional synthetic plastics [59]. Bioplastics, derived from renewable sources such as starch, cellulose, and polylactic acid (PLA), are increasingly being used to address sustainability challenges due to their biodegradability, lower carbon footprint, and reduced energy consumption during production [60]. To enhance the functionality of these materials, functional nanomaterials are being incorporated into biopolymer matrices, significantly improving mechanical strength, barrier properties, moisture resistance, and flexibility [61]. The addition of montmorillonite clay NPs to PLA improves its gas barrier properties and tensile strength, making it more suitable for perishable food packaging [62]. Nanocellulose and nano-clay enhance the mechanical strength and moisture barrier properties of biodegradable films, enabling their use as sustainable alternatives to synthetic plastics. Their biocompatibility supports eco-safe applications in active or intelligent formats [63,64]. The development of protein-derived nano-biocomposites using layered silicates or other nanomaterials has become a promising strategy for creating multipurpose packaging materials. These composites demonstrate improved thermal stability, UV resistance, and oxygen barrier performance, positioning them as competitive alternatives to traditional petroleum-based plastics [65,66]. Despite these advances, active bio-based packaging that interacts with food to extend shelf life or monitor quality remains relatively rare. However, innovative approaches are emerging. Recent research has focused on developing smart dopamine-functionalized biopolymer NPs that exhibit pH sensitivity, ratiometric fluorescence, and photocatalytic behavior, making them suitable for incorporation into packaging films that indicate food spoilage through color changes [67,68]. These NPs can be immobilized into paper or polymer matrices using matrix solid-phase dispersion techniques, creating smart packaging capable of non-destructive, real-time food quality monitoring. Embedding food quality indicators in biopolymer-based nanocomposites can reduce variability and enhance stability [69,70]. Curcumin-loaded NPs have been incorporated into gelatin films to provide colorimetric responses to pH changes in seafood packaging, enabling users to detect spoilage without opening the package [71]. Such systems offer a bio-based platform for intelligent packaging that maintains compatibility with existing manufacturing processes, enabling a smooth transition from conventional to innovative, sustainable packaging [26]. Biopolymer-based nanocomposites represent a new generation of food packaging materials that are renewable, sustainable, and functional [72]. They can preserve food quality by limiting undesirable interactions, blocking mass transfer of gases and vapors, filtering UV radiation, and enabling embedded sensor technology for real-time quality assessment [73]. Table 1 summarizes the four major categories of smart food packaging, highlighting their key characteristics, functional mechanisms, and common food industry applications.

## 3. Nano-Enabled Sensing Mechanisms and Functional Applications in Smart Food Packaging

**Figure 2:**
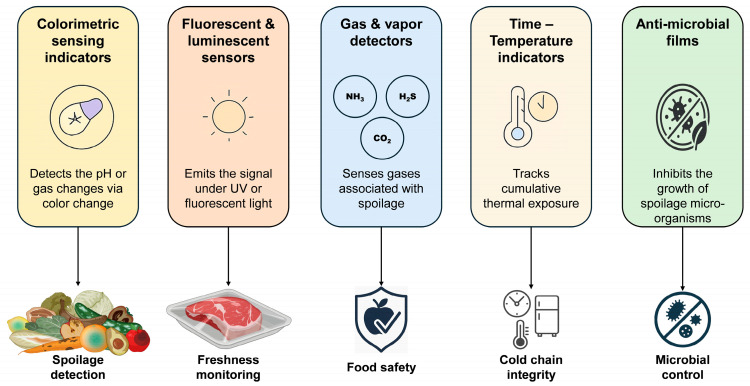
Nano-enabled sensing technologies in smart food packaging and their primary function.

Ensuring food safety, extending shelf life, and maintaining product integrity are central goals in the modern food industry. The emergence of nano-enabled smart food packaging provides a breakthrough in achieving these goals by offering real-time monitoring, active spoilage prevention, and improved consumer transparency. This section outlines the primary sensing mechanisms and practical applications of nanomaterials incorporated into smart packaging systems. Figure 2 illustrates the various nano-enabled sensing mechanisms, including colorimetric, fluorescent, gas sensing, time–temperature indicators, and antimicrobial films, used in smart packaging to enhance spoilage detection, freshness monitoring, food safety, and microbial control.

### 3.1. Colorimetric Sensing Indicators

Colorimetric indicators provide a simple, visual representation of food quality changes, primarily by detecting shifts in pH or volatile gas emissions during spoilage. These systems often use natural pigments (e.g., anthocyanins, curcumin) or pH-responsive dyes, which undergo visible color changes in response to chemical stimuli [74,75]. For instance, anthocyanin-based films extracted from red cabbage have been incorporated in biodegradable matrices to monitor freshness in meat and seafood products [34,76]. Metal oxide NPs, such as ZnO, have also been incorporated into colorimetric systems to provide low-cost, pH-sensitive spoilage detection [75]. At the nanoscale, ZnO NPs or anthocyanin-loaded films exhibit enhanced sensitivity due to increased surface activity and their ability to act as catalysts in acid–base reactions. This amplifies visible color changes under minimal pH or gas fluctuations, allowing earlier spoilage detection than bulk materials [77].

### 3.2. Fluorescent and Luminescent Sensors

Fluorescent indicators utilize nanocomposite materials containing fluorophores or quantum dots that emit measurable signals upon interacting with spoilage-related molecules such as hydrogen sulfide, fatty acids, or volatile amines [76,78]. Beyond visible color changes, fluorescence-based indicators offer enhanced sensitivity and can detect early-stage spoilage events. Smart films embedded with fluorescent ZnO nanocomposites or organic luminophores produce luminescent signals that can be visually assessed or detected using optical devices under UV illumination [79,80]. Such systems enhance user-friendliness and allow rapid, non-destructive monitoring. Quantum dots and carbon dots exhibit size-dependent quantum confinement effects, which lead to sharp emission spectra and tunable fluorescence properties. This makes them ideal for detecting microbial metabolites or biogenic amines via fluorescence quenching or enhancement in smart packaging [81].

### 3.3. Gas and Vapor Detectors

Gases like ammonia (NH_3_), hydrogen sulfide (H_2_S), carbon dioxide (CO_2_), and ethylene are common spoilage by-products. Gas sensors embedded in packaging detect these volatiles using nanomaterials such as metal oxides (e.g., SnO_2_ doped with Ag or Co), conductive polymers, or carbon-based nanostructures like MWCNTs and graphene [82,83,84]. Nanomaterials offer increased surface reactivity and selectivity, making them ideal for real-time freshness tracking. These gas sensors have been shown to significantly reduce spoilage and enhance food safety by offering precise environmental monitoring within the package [28,85]. Metal oxide NPs (e.g., SnO_2_, ZnO) operate on the basis of chemisorption of target gases like NH_3_ or H_2_S. The adsorbed gas alters the surface charge density, modulating electrical resistance. Their nanoscale morphology enhances surface-to-gas interaction, improving detection accuracy and response time [86].

### 3.4. Time–Temperature Indicators (TTIs)

TTIs record the cumulative thermal exposure of food products, making them indispensable in cold chain logistics. Nano-enabled TTIs typically use temperature-responsive dyes or materials embedded in biopolymer matrices (e.g., CMC or PLA) that undergo irreversible color changes [87,88]. A notable example is polydiacetylene (PDA)/Ag NPs composites embedded in CMC films. These systems show color transitions (e.g., purplish-blue to reddish-purple) in response to specific thermal events, indicating potential spoilage risks [15]. Further innovations include programmable LED-based TTIs triggered by heat changes, enhancing supply chain traceability [89]. PDA/Ag NPs exhibit phase transitions or plasmonic shifts under cumulative thermal exposure. These shifts result in irreversible color changes that serve as visual thermal history indicators mainly for highly perishable foods [15,90].

### 3.5. Spoilage and Pathogen Detectors

Spoilage detectors work by sensing physical or chemical markers such as pH shifts, odors, or enzymatic activity linked to microbial contamination [32,91,92]. Electrochemical probes, biosensors with antibodies or aptamers, and fluorescence-based pathogen detectors have been embedded into nanostructured films to allow real-time, sensitive microbial monitoring [75,93,94,95]. Nanoscale biosensors utilize immobilized aptamers or antibodies on conductive nanomaterials (e.g., graphene, CNTs), where pathogen binding causes measurable changes in current or fluorescence due to electron transfer disruptions at the nanoscale interface [95].

### 3.6. Functional Application: Antimicrobial Films

Antimicrobial packaging prevents microbial proliferation by incorporating agents that inhibit bacterial and fungal growth. Silver (Ag), copper (Cu), and zinc oxide (ZnO) NPs are widely used for their broad-spectrum antimicrobial properties, particularly in room-temperature storage [93,96]. Ag-coated bacterial cellulose films, for example, exhibit potent activity against foodborne pathogens [97]. Biodegradable polymers such as polylactic acid (PLA) and thermoplastic starch (TPS), although inherently weak in barrier performance, can be reinforced with nano-clays or cellulose nanocrystals to form durable, reusable, and edible antimicrobial films [26]. These films exploit the nanoscale release kinetics of Ag, Cu, or ZnO ions, enabling prolonged antimicrobial action by maintaining a controlled release profile [98]. Table 2 summarizes the nano-enabled sensing mechanisms used in smart food packaging systems, highlighting the key sensing principles, nanomaterials employed, target analytes, and their typical food applications. These systems enhance food safety, freshness monitoring, and spoilage detection through various physicochemical interactions.

## 4. Advanced Food Packaging Materials with Nano-, Bioactive, and Biopolymer Compounds

**Figure 3:**
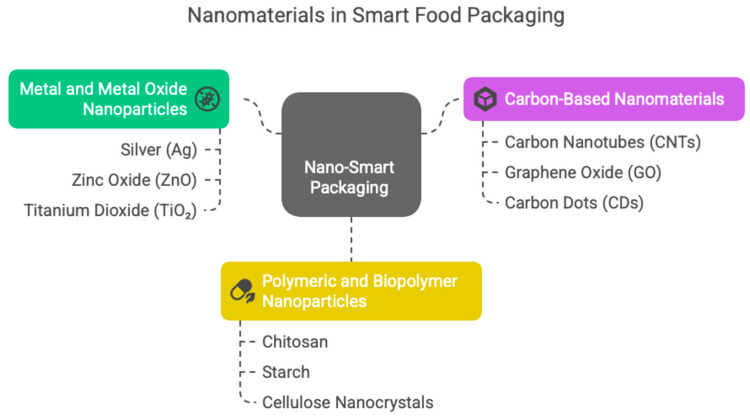
Primary categories of nanomaterials commonly used in nano-smart packaging.

Incorporating nanomaterials has revolutionized smart food packaging by enhancing its functionality, responsiveness, and sustainability [10,20]. Their unique properties, including a high surface area and antimicrobial activity, make them ideal for the development of advanced packaging systems [10]. Nanocomposites that incorporate various NPs can improve barrier properties, mechanical strength, and antimicrobial efficacy, thereby extending shelf life and ensuring food safety [93]. These materials facilitate real-time monitoring through biosensors and IoT integration, thereby enhancing traceability and reducing waste [20]. Recent developments focus on biodegradable nanocomposites to address environmental concerns [99]. However, challenges persist regarding regulations, sustainability, and consumer acceptance [20]. Despite these hurdles, nanomaterial-based smart food packaging offers significant potential for improving food quality, safety, and sustainability in the food industry [10,93]. This section discusses three major categories of nanomaterials commonly used in nano-smart packaging: metal and metal oxide NPs, carbon-based nanomaterials, and polymeric/biopolymer NPs (Figure 3).

### 4.1. Metal and Metal Oxide Nanoparticles

A wide variety of nanocomposites are being developed and applied in food packaging due to their remarkable antimicrobial, mechanical, and barrier properties. Metal and metal oxide NPs like silver (Ag), zinc oxide (ZnO), titanium dioxide (TiO_2_), and copper oxide (CuO) have gained significant attention in smart food packaging due to their multifunctional properties. These M-NPs enhance mechanical performance, barrier properties, and extend the shelf life. It can provide antimicrobial and antioxidant activities when incorporated into biopolymers and petroleum-based polymers [100,101]. The addition of M-NPs improves temperature and moisture stability, enhances UV protection, and contributes to reduced ethylene and oxygen exposure through mechanisms such as gas adsorption, catalytic degradation, or barrier property enhancement [100]. Inorganic M-NPs in packaging materials reduce bacterial growth and waste generation during the packaging process [102]. Smart packaging solutions utilizing M-NPs can also act as freshness indicators through pH-sensitive or color-changing mechanisms [103].

However, potential particle migration to food and the environment, as well as toxicological effects, remain essential considerations in the development of M-NP-based packaging systems [100,101]. In the case of nano-Ag, which is synthesized chemically, it is widely used in packaging films and coatings for its strong ability to prevent microbial contamination and spoilage, effectively extending shelf life [104]. Similarly, ZnO NPs exhibit both antimicrobial and antioxidant properties and are often used as barriers against gases and moisture in food packaging [105]. TiO_2_ NPs, also chemically synthesized, offer photocatalytic activity and enhance mechanical strength, making them suitable for active food packaging applications [104]. CuO NPs, recognized for their potent antimicrobial properties, help in extending product freshness and are utilized in intelligent packaging systems [106]. In contrast to chemically synthesized nanomaterials, green synthesis methods are gaining popularity due to their environmental and safety advantages. Nanocrystalline cellulose (NCC) combined with metal NPs also presents a green approach, providing superior mechanical and barrier properties as well as antimicrobial activity, and is especially valuable in developing biodegradable and advanced smart packaging systems [107]. Gold NPs, which can be synthesized chemically or via green routes, show antioxidant and mild antimicrobial properties and are commonly applied in intelligent packaging, particularly as color indicators, though their use is limited by high cost and lower prevalence [105,108]. More complex composites, such as nanoiron-based materials like zero-valent iron embedded in polymers, exhibit high oxygen reactivity and are used as oxygen scavengers to extend shelf life and preserve food quality [109]. An innovative example is the Fe_3_O_4_–cellulose nanofiber aerogel infused with thyme essential oil, which combines antibacterial and magnetic responsiveness with a controlled release of active compounds, making it effective for prolonging the shelf life of meat and fruits while preserving sensory qualities [110]. Magnesium oxide NPs (MgONPs), produced through physical, chemical, or green synthesis methods, offer thermal stability, UV shielding, antimicrobial activity, and excellent compatibility with natural polymers such as chitosan and starch. These characteristics make MgONPs ideal for biodegradable active packaging [111]. MgONPs synthesized via physical, chemical, or green synthesis have intrinsic antimicrobial activity due to their ability to generate reactive oxygen species (ROS) and disrupt microbial membranes [112]. Their high thermal stability and UV shielding properties help to preserve food quality by inhibiting photo-degradation and thermal oxidation. Additionally, their surface charge and biocompatibility with natural polymers such as chitosan and starch allow uniform dispersion, which enhances mechanical integrity and barrier properties [113]. These synergistic features make MgONPs particularly effective for use in biodegradable active packaging systems. Other advanced nanocomposites include nanosilica/polymer hybrids, which improve mechanical strength, thermal stability, and moisture/gas barrier functions, enhancing shelf life in smart packaging systems [114]. Lastly, aluminum oxide NPs integrated into sol–gel coatings on PET films enhance gas barriers, puncture resistance, and heat-sealability while maintaining transparency and recyclability, representing a sustainable solution for modern food packaging [115]. Table 3 summarizes an overview of nanomaterials used in advanced food packaging systems, including their synthesis method, properties, and typical applications. These materials enhance antimicrobial efficacy and barrier performance, with intelligent packaging functionalities.

Furthermore, metal NP-based sensors offer innovative solutions for detecting food spoilage and ensuring food safety. These M-NPs can act as sensitive nanosensors for detecting gases and volatile organic compounds (VOCs) released during food spoilage. For instance, Ag-doped ZnO NPs have demonstrated excellent ethanol vapor sensing capabilities, with optimal performance at 320–325 °C [128]. TiO_2_-coated films can effectively inactivate various microorganisms, including *E. coli*, *S. aureus*, and fungi [129,130]. The antimicrobial activity increases with higher TiO_2_ concentrations and longer light exposure, with UV light being more effective than fluorescent light [130]. Ag and Au NPs embedded in bacterial cellulose or paper substrates can act as plasmonic nanosensors, changing color in response to volatile compounds released during food spoilage [90,131]. These sensors exhibit high sensitivity and selectivity to biogenic amines and other spoilage indicators, while remaining unaffected by common gases like CO_2_ or water vapor [131]. These nanosensors can be integrated into smart packaging systems to monitor food quality throughout the supply chain [80]. The unique properties of nanomaterials enable the creation of highly sensitive and selective sensors for detecting contaminants, heavy metals, and pathogens in food and agricultural applications [132].

### 4.2. Carbon-Based Nanomaterials

Carbon-based nanomaterials (C-NPs), including carbon nanotubes (CNTs), graphene oxide (GO), and carbon dots (CDs), have emerged as promising candidates for smart packaging applications due to their exceptional properties [133,134]. These materials offer high sensitivity and selectivity in detecting food spoilage markers and adulterants through various sensing mechanisms, such as electrochemical and fluorescence-based sensors [135]. Their large surface area, functionality, and mechanical stability make them ideal for developing advanced sensors for food safety and quality control [133]. Recent advancements in C-NP-based sensors have focused on detecting spoilage pathogens, toxins, pH changes, and gases in food products [135]. Additionally, these nanomaterials have been incorporated into pH sensors and time–temperature indicators for real-time monitoring of food quality, addressing the global issue of food wastage [28]. This is due to their high surface area, π-conjugated structure, and electrical conductivity, which facilitate strong analyte interactions, rapid signal transduction, and high sensitivity in electrochemical and fluorescence-based sensors [136]. These properties allow for real-time, low-threshold detection of spoilage indicators in perishable food products. Carbon dots (CDs) are emerging nanomaterials with unique properties that make them promising for food packaging applications [137]. They can be synthesized from sustainable sources like food waste and by-products, aligning with circular economy principles [138,139]. CDs enhance packaging materials’ mechanical, barrier, and preservative properties, functioning as antioxidants, antimicrobials, and UV blockers [138,140]. Their low toxicity and biocompatibility make them suitable for active and intelligent packaging systems [140,141]. CDs can be incorporated into films to create multifunctional packaging materials that extend foods’ shelf life and improve safety [141]. The synthesis of CDs from food waste and cooking processes offers a sustainable approach to producing these valuable NPs [139,141].

Recent research demonstrates the potential of graphene oxide (GO) in enhancing food packaging materials. GO can be incorporated into various polymer matrices, including gelatin and poly(lactic acid), to improve barrier properties against oxygen and moisture [142,143]. These nanocomposites exhibit enhanced mechanical strength, thermal stability, and antimicrobial properties [144]. Notably, reduced GO films as thin as 30 nm can provide an impermeable barrier to gases and liquids, including aggressive chemicals [145]. The addition of GO to packaging materials significantly decreases oxygen permeability, with some studies reporting reductions of up to 99.3% compared with unmodified films [142]. Furthermore, GO-enhanced films maintain good transparency and mechanical properties, making them suitable for food packaging applications [142,143].

### 4.3. Polymeric and Biopolymer Nanoparticles

Polymeric and biopolymer NPs such as chitosan, starch, and cellulose nanocrystals are increasingly used in smart packaging due to their biodegradability and compatibility with food products. These nanomaterials can be engineered to carry bioactive compounds (e.g., antioxidants, antimicrobials) and sensing agents for intelligent food packaging systems [8,146]. These P-NPs enhance the mechanical, thermal, and barrier properties of packaging films while maintaining biodegradability [8]. Starch, chitin, chitosan, and alginate NPs are non-toxic, antimicrobial, and excellent candidates for nano-reinforcements in bio-nanocomposites. For example, chitosan possesses inherent antimicrobial properties and can be combined with NPs like titanium dioxide to enhance its effectiveness in preserving food [40,147]. These nanocomposites can extend the shelf life of various food products, including fruits, vegetables, meat, and dairy [40]. Nanoencapsulation of active compounds like anthocyanins, essential oils, and antimicrobials can improve the functional performance of packaging materials, enabling features such as light blocking, freshness monitoring, and controlled release of active ingredients [69]. The integration of nanotechnology with biopolymers offers promising solutions for sustainable and intelligent food packaging applications. Biopolymer-based nanocomposites (BP-NPs) offer improved mechanical properties and environmental sustainability compared with conventional plastics. These eco-friendly materials incorporate nano-sized bio-based reinforcements into biopolymers like polylactic acid, starch, and chitosan [148]. Incorporating BP-NPs like nano-clay or nanocellulose into biopolymers enhances tensile strength, flexibility, and thermal stability [148,149]. BP-NPs also increase water resistance and reduce gas permeability, making them suitable for packaging applications [149]. The addition of nanocellulose to biopolymer matrices like starch, chitosan, and polylactic acid improves mechanical strength and oxygen–water vapor barrier properties [150]. From a sustainability perspective, nano-clay production results in lower energy use and greenhouse gas emissions compared with many common biopolymers and glass fibers [151]. However, challenges remain, including poor dispersion of nano-reinforcements and sensitivity to moisture and temperature [148]. Within the framework of promoting a greener and more sustainable environment, biopolymers (such as starch, cellulose, chitin, chitosan, zein, and gelatin) have emerged as alternatives to synthetic polymers [152]. However, biopolymer-based food packaging has its limitations, including poor mechanical, thermal, barrier [153], and hydrophilic properties [100]. Therefore, research initiatives have been increasingly directed toward enhancing their quality and performance [154]. Incorporating NPs into biopolymers modulated a range of physiological and biological properties. Studies have shown that NP-based biopolymers enhanced mechanical and barrier properties [155]. Among NPs that have been conjugated with biopolymers, metals such as silver, copper, and gold, along with metal oxides (ZnO, TiO_2_, MgO, Ag_2_O), have been used for food packaging [153]. These metal NPs demonstrated their ability to improve the functional properties of food packaging materials and monitor food quality [100]. In particular, the mechanical characteristics of the biopolymers depend on the nature of the incorporated NPs. For instance, Ag NPs incorporated in shrimp chitosan-based edible films produced a stronger and more elastic material [156]. In contrast, the addition of ZnO NPs to biopolymers reduced the tensile strength and elastic modulus of the films. This effect can be related to the weak interfacial interaction between the polymer matrix and ZnO NPs [157]. Concerning the antibacterial properties, NPs exhibited a significant effect against various microbial strains, proving their efficiency for becoming an active packaging film. The efficiency of Ag NPs toward Gram-positive bacteria (*S. aureus*,) when embedded in hydrogels (chitosan and hydroxypropyl methylcellulose) [158] or starch–gelatin hydrogel [159] was proved. In addition, the incorporation of Ag NPs also induces a synergistic activity toward *Botrytis cinerea* that increases the inhibition capacity of the chitosan films [156]. On the other hand, ZnO NP-impregnated films inhibited the growth of *L. monocytogenes* and *E. coli* [157]. While these metal NPs present considerable benefits, they pose concerns regarding their leaching and migration into food [100]. Consequently, natural nano-based filler materials can serve as an effective alternative. Promising results were obtained using cellulose nanofibers isolated from unripe banana peel, as evidenced by the improvement in barrier, optical, and mechanical properties, producing a value-added material [160]. Similarly, cellulose nanowhiskers extracted from mulberry pulp, when blended with alginate, enhanced the mechanical properties of alginate films by improving the tensile strength with a low concentration of cellulose nanowhiskers (up to 4 wt%) [161]. The study of gelatin/zein nanofibers co-loaded with cinnamaldehyde–thymol showed great antibacterial activity against *E. coli*, *S. aureus*, and *L. monocytogenes*, as well as good antioxidant ability [162]. The incorporation of NPs in biopolymers gives a new direction in the production of food packaging. Notably, several properties of the biopolymer were improved. However, ensuring safety and preventing the migration of the NPs to the food remains a critical consideration that require further investigations. Amid these concerns, natural NPs have emerged as a promising and safe material capable of ensuring both safety and quality.

### 4.4. Integration with Bioactive Compounds

The growing need for eco-friendly and sustainable food packaging choices that provide additional health advantages has led to a rise in interest in active packaging techniques in recent years [163]. Among the various strategies, one involves the incorporation of secondary metabolites, known as bioactive compounds, which are found in minimal concentrations in several plant species [164]. They are used in food packaging in several ways, such as incorporating these phytochemicals into natural biopolymers to remove chemical contamination from food [163]. Recent research has focused on nanotechnology techniques in order to improve the effectiveness of these compounds, like nanoencapsulation and electrospinning [165]. Encapsulation is a promising technique to protect the bioactive compounds (e.g., antioxidants and antimicrobials) from chemical and thermal degradation to improve their stability, bioavailability, and solubility, besides regulating the release of these active substances [166]. Electrospinning is an effective and adaptable electrodynamic method for producing continuous nano-sized fibers from a range of polymeric materials. The electrospun nanofibrous materials exhibit exceptional characteristics such as elevated porosity, extensive specific surface area, and superior load efficiency [167]. This technique can be used to generate nanofibers for the encapsulation of unstable bioactive molecules and the incorporation of NPs [168], or to produce nanofibers designed for the controlled release of bioactive compounds [169]. Polyphenolic compounds and essential oils are among the most prevalent bioactive substances utilized in active food packaging [8,9]. Natural phenolic compounds are excellent options for food packaging due to their antibacterial and antioxidant properties, along with other important features like color, flavor, UV light resistance, and sensory appeal [170]. For instance, green tea extract rich in phenolic compounds such as catechins (e.g., epigallocatechin gallate) was successfully integrated into polyvinyl alcohol (PVA)-based nanofibers using electrospinning by Alav et al. in 2024 in order to improve microbiological stability and increase the shelf life of kiwifruit [171]. On the other hand, essential oils are volatile mixtures that exhibit good antioxidant and antimicrobial activities, helping improve the shelf life [8,11]. Centrifugally spun gelatin-based fibers containing bay laurel leaf essential oil were designed and refined by Guler et al. (2024) for use in active packaging, as they showed good antioxidant and antimicrobial activity [172]. Similarly, using the cutting-edge electrospinning method, chitosan/(PVA) hybrid nanofibers containing three different essential oils (lemon, lime, and grapefruit) were effectively produced. The resultant nanofibers showed noteworthy antibacterial, tyrosinase inhibitory, and antioxidant properties. They effectively preserved the quality of citrus essential oils and extended their applicability in active food packaging [173]. Table 4 summarizes examples of nanotechnology-based techniques for incorporating bioactive compounds into active food packaging.

## 5. Safety, Toxicity, and Regulatory Aspects

**Figure 4:**
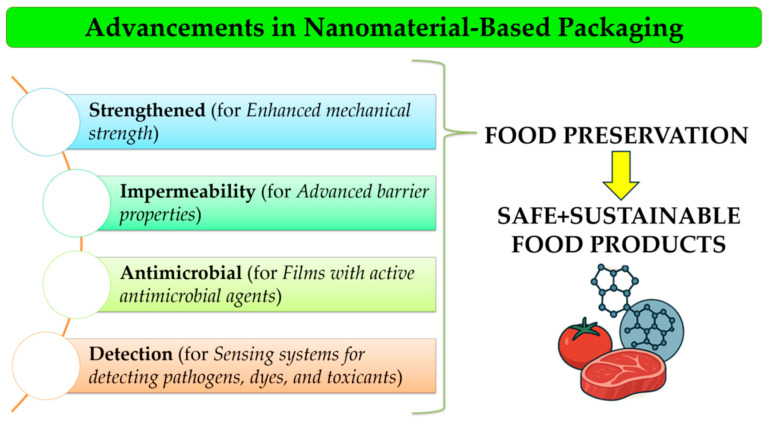
Advantages of the application of nanotechnology in food packaging.

Incorporating nanomaterials into food packaging can enhance food preservation, storage, distribution, and human food consumption [182,183]. Food packaging is a barrier to limiting components’ migration and slowing product deterioration in food processing and storage. Conventional polymer films, such as polyolefins, ethylene copolymers, and polyesters, are widely used [184], permeating gases like water vapor, oxygen, and carbon dioxide. All of these limit their protective capacity, so it should be improved. For problem-solving management, fillers or plasticizers can be integrated into polymer-based products, strengthening their barrier capabilities and reducing microbial contamination [93]. Furthermore, studies show the importance of nanomaterials with nanoscale dimensions and the ability to carry bioavailable nutrients [184,185]. Nanotechnology-enhanced packaging offers superior gas and moisture resistance, enhanced mechanical properties, antimicrobial activity, and even innovative functionalities, such as gas sensing (Figure 4) [2,8,9,10]. Several nanomaterials can be additives that significantly elevate packaging films’ mechanical, chemical, and barrier qualities, even at low loadings [186,187]. Specific metal-based NPs including silver (Ag), copper (Cu), zinc oxide (ZnO), and titanium dioxide (TiO_2_) can demonstrate broad-spectrum antibacterial activity through multiple mechanisms, such as damaging microbial cell membranes, producing reactive oxygen species (ROS), and releasing metal ions that interfere with essential cellular functions [188,189]. Potential nano-packaging materials, like carbon-based nanomaterials, including carbon nanotubes (CNTs) and graphene oxide (GO), can support the packaging film’s mechanical strength and barrier properties.

This fact can be discussed alongside the high aspect ratios and strong interfacial interactions with polymer compounds that contribute to improved tensile strength and reduced permeability to gases and moisture [190,191]. Spoilage reduction can be achieved through the protective characteristics of fibrous nanomaterials [104]. For instance, electrospun nanofibers can form mats or coatings as physical barriers against microbial invasion [192]. These can be integrated into packaging to create active layers that protect against spoilage and can be functionalized with antimicrobial agents for enhanced protection [193]. In the realm of sustainable packaging, polysaccharide-based nanofillers, including cellulose nanocrystals (CNCs) and chitosan NPs, offer biodegradable and renewable options for packaging enhancements. This can be due to their rigid crystalline structure, ability to enhance barriers [194], and ability to be loaded with bioactive compounds [195]. Furthermore, functionalizing NPs with bioactive agents, such as phytochemicals, can significantly improve packaging materials’ antimicrobial and antioxidant properties. Phytochemicals like polyphenols and flavonoids can be conjugated onto NPs’ surfaces, leading to synergistic antimicrobial effects [196] and controlled release [197]. Despite these advancements, it is essential to evaluate the interactions between nanomaterials and food components thoroughly. High concentrations of antimicrobial agents, if not carefully controlled, may negatively impact food’s sensory qualities, safety, or nutritional profile. To address these concerns, controlled release systems are increasingly being explored to ensure long-term antimicrobial activity without compromising food integrity [198,199]. Nevertheless, notable knowledge gaps remain. While substantial research has addressed the physical, chemical, and microbiological implications of nano-enabled packaging, the role of organic nanofillers and their long-term behavior, especially in direct contact with complex food matrices, is still insufficiently understood. Integrating nanomaterials in food packaging raises safety issues, mainly concerning whether NPs can migrate into food when exposed to harsh conditions like high humidity, acidity, or high temperatures [200]. Ingesting NPs, especially those smaller than 10 nm, might cause effects such as cytotoxicity, genotoxicity, or bioaccumulation, as they can penetrate cells and interact with organelles [201,202]. Although some research shows risks like oxidative stress or DNA damage, long-term toxicological data are still lacking. The varying properties of NPs make safety assessments more complicated. Agencies like the EFSA and FDA recognize these risks but do not have widely accepted testing standards [203]. Limited labeling and transparency contribute to public skepticism, highlighting the need for effective risk communication, reliable migration models, and clear regulations to ensure safe use [204]. Further in-depth investigations are needed to explore their interactions, migration potential, and overall safety, which is essential for ensuring safe and appropriate application in nanomaterial-based food packaging systems [204,205].

## 6. Challenges and Future Directions for Nano-Enabled Food Packaging

Nanotechnology holds great potential in the food sector through enhanced safety measures, prolonged product shelf life, improved nutritional content, and promoting sustainable agricultural practices, drawing considerable interest from researchers and policymakers [206,207]. However, several challenges (seen in Table 5) must be addressed to ensure its safe and practical implementation.

**Figure 5:**
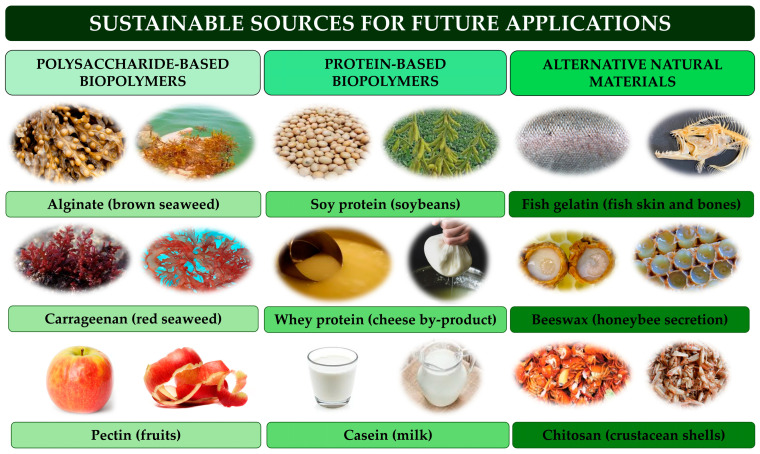
Green sources for food packaging nanomaterials.

Currently, conventional plastics dominate the food packaging market, contributing significantly to environmental degradation due to their non-biodegradable nature. This challenge has accelerated the development of sustainable packaging alternatives sourced from renewable materials [44,213]. Biodegradable polymers such as starch, cellulose, polylactic acid (PLA), and polyhydroxyalkanoates (PHA) are increasingly favored for their renewability, biodegradability, and lower environmental impact. These properties can be further enhanced by incorporating nanomaterials, inorganic fillers, or plasticizers, thereby improving mechanical strength and barrier efficiency and enhancing their suitability for packaging applications [214,215]. Natural polymers, including polysaccharides (e.g., alginate, carrageenan, pectin) and proteins (e.g., soy, whey, casein), are especially promising due to their edibility, biodegradability, and film-forming capabilities [216,217]. Sustainable alternatives, like fish gelatin, beeswax, and chitosan, are also under continuous research and development [218,219,220]. They offer an eco-friendly and safe alternative for food packaging. However, their natural permeability to moisture and gases may require enhancement [221]. Some examples of the sources of these biopolymers are presented in Figure 5. 

**Figure 6:**
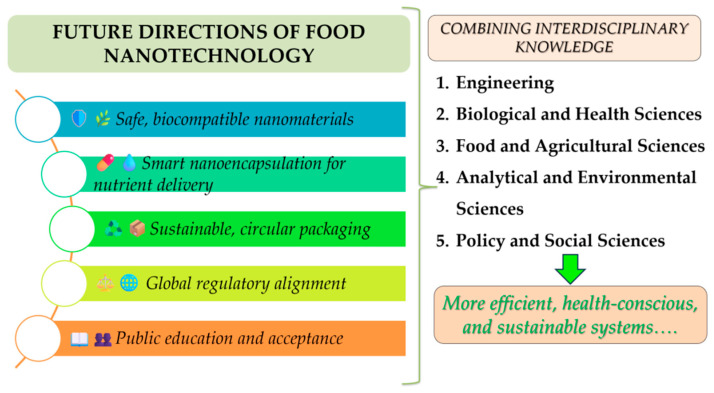
The role of nanotechnology in the future of food packaging.

To fully realize the benefits of nanotechnology in food systems, an interdisciplinary approach that bridges, for instance, materials science, toxicology, food engineering, and regulatory policy is essential [222,223,224]. Figure 6 summarizes some aspects of the future directions. By aligning technological innovations with policy frameworks and public communication strategies, nanotechnology can transition from laboratory innovation to market-ready applications [225], tackling critical global challenges like ensuring food safety [226] and promoting environmental sustainability [227].

Incorporating nanomaterials can be a transformative opportunity for sustainable food packaging; its success hinges on resolving scientific, regulatory, and societal challenges. A collaborative, interdisciplinary approach involving toxicologists, material scientists, industry stakeholders, and regulators is key to safely advancing these innovations from the lab to the market.

## 7. Conclusions

The global food industry is working to improve the quality, safety, and shelf life of food products while reducing packaging waste and its environmental impact. In this effort, nanomaterials have become crucial for creating smart and sustainable food packaging systems. Their unique properties, such as large surface areas, adjustable reactivity, antimicrobial effects, and enhanced barrier performance, allow for the design of packaging that is both protective and interactive. Bio-based nanomaterials, which naturally break down and can be reused, offer a promising alternative to conventional plastics. When combined with biopolymer matrices, nanomaterials significantly enhance the physical and mechanical properties of packaging, including strength, gas barrier efficiency, and heat resistance. This review also highlights the potential of smart nanomaterials, such as metal NPs, carbon-based materials, and biopolymeric NPs, to enable real-time freshness detection, gas sensing, and environmental responsiveness. The development of active and intelligent packaging that can interact with food or its environment opens new possibilities for food safety, traceability, and consumer engagement. These smart systems can be further improved by integration with other preservation strategies, such as modified atmosphere packaging, biosensors, and cold chain technologies, particularly for lightly processed and ready-to-eat products. However, despite their great potential, several challenges must be addressed. The small size and high reactivity of specific nanomaterials raise concerns about their migration into food and potential long-term effects on human health and the environment. Unclear regulations, especially in developing countries, further complicate the path to commercialization. Establishing standardized risk assessment protocols and safety evaluation criteria is crucial for ensuring consumer safety and maintaining public trust. Additionally, limited access to advanced technologies, funding, and infrastructure remains a challenge for small and medium-sized enterprises (SMEs) involved in nanotechnology-based food packaging. To support widespread industrial adoption, future strategies must focus on managing waste throughout the product lifecycle, developing scalable green synthesis methods, and affordable manufacturing processes. Looking ahead, combining nano-enabled packaging with biodegradable films, edible coatings, biosensing platforms, and recyclable systems will play a critical role in reducing food waste, enhancing product safety, and achieving sustainability goals. As the threat of foodborne contamination and global supply chain disruptions grows, the demand for intelligent packaging designed to detect and respond to long-term pathogens will only increase. It is expected that international regulatory standards will evolve better to accommodate the rapid innovation in nano-enabled food packaging. Collaboration across disciplines, including materials science, food technology, toxicology, and policy, will be key to unlocking the full potential of smart nanomaterials, ensuring that these innovations contribute meaningfully to public health, environmental protection, and global food security.

## Figures and Tables

**Table 1:** Classification of different smart food packaging systems and its applications.

Packaging Type	Key Features	Mechanism of Action	Applications	Nanomaterials Used
Active packaging	Interacts with food or the environment	Releases/absorbs substances (e.g., oxygen, moisture)	Antimicrobial films, oxygen scavengers	Nano-Ag, CuO, ZnO, nano-clay
Intelligent packaging	Monitors the condition of food/environment	Colorimetric sensors, gas indicators, TTIs	Freshness indicators, spoilage detection	Quantum dots, ZnO, CNTs, CDs
Interactive packaging	Digitally enhanced, provides data access	QR codes, RFID, NFC technologies	Supply chain traceability, authentication	CNTs, graphene, nanocomposite inks
Bio-based packaging	Derived from renewable sources, biodegradable	Integrates nanomaterials in biopolymer matrices	Edible films, compostable nanocomposites	CNCs, nano-starch, layered silicates

**Table 2:** Comparative analysis of nano-enabled smart packaging sensors.

Mechanism	Sensing Principle	Nanomaterials Used	Target Analyte	Application
Colorimetric	pH/gas-triggered dye change	ZnO (with anthocyanins as colorimetric agent),	Volatile amines	Spoilage detection in meat
Fluorescence-based	Emission under UV light	Quantum dots, CDs, ZnO	Microbial metabolites	Dairy spoilage sensors
Electrical conductivity	Resistance/capacitance shift	CNTs, graphene, metal oxides	Pathogens, gases	Sensor chips in seafood packs
Gas sensing	Chemisorption	SnO_2_, MWCNTs, Ag-doped ZnO	NH_3_, H_2_S, CO_2_	MAP packaging freshness tracking
Time–temperature	Thermal response of indicators	PDA/Ag NPs	Cumulative heat	Cold chain integrity

**Table 3:** Nanomaterials for advanced smart food packaging and its applications.

Nanomaterial Used	Method of Synthesis	Properties	Applications	Ref.
Ag	Chemical	Antimicrobial and extends shelf life	Milk storage	[116]
Ag	Green	High H_2_S sensitivity	Chicken breasts, and fish fillets	[117]
Au	Chemical	Visual color indicator	Fish fillets	[118]
Au/Ag	Chemical	Antimicrobial, antioxidant, and better mechanical properties	Cheddar cheese	[119]
TiO_2_/Ag	Green	Antimicrobial and UV blocking	Cherry tomatoes	[120]
TiO_2_	Chemical and electrospinning	Photocatalytic degradation and extends shelf life	Bananas	[121]
Fe_2_TiO_5_	Sol–gel	Antimicrobial, no cytotoxicity, and shelf life	Strawberries	[122]
Cu	Solvothermal	Antimicrobial and antioxidant	Shrimp	[123]
ZnO	Chemical	Antimicrobial, antioxidant, and extends shelf life	Pork meat	[124]
ZnO	Hydrothermal	Improved mechanical strength and antimicrobial	Black grapes	[125]
ZnO-Fe_2_O_3_	Chemical	Antimicrobial and shelf life	Tomatoes	[126]
Se^0^	Chemical and green	Improved mechanical properties, antimicrobial, and antioxidant	Beef	[127]

**Table 4:** Overview of bioactive compounds incorporated into food packaging materials using nanotechnology techniques.

Bioactive Compound	Polymer Used	Method of Synthesis	Functional Property	Ref.
*A. yomena* extract	Zein-polycaprolactone	Electrospinning	Antioxidant and antibacterial against *E. coli* and *B. subtilis*	[174]
Tea polyphenol	PVA/ethyl cellulose	Electrospinning	Antioxidant and antimicrobial activity against *E. coli* and *S. aureus*	[167]
Jujube extract	PVA	Electrospinning	Antioxidant and antibacterial	[175]
Cardamom essential oil	CMC	Nano-emulsion	Antibacterial and antibiofilm action against *E. coli* and *L. monocytogenes*	[176]
Carvacrol	Gelatin/chitosan	Electrospinning	Antioxidant and antibacterial activity against *E. coli* and *S. aureus*, sustained release of carvacrol	[177]
Thymol	CS/PVA	Encapsulation and film incorporation	Antibacterial activity against *E. coli* and sustained release of thymol	[178]
Oregano essential oil	PLA–PCL	Electrospinning	Antibacterial and antifungal activity	[179]
Cinnamon–perilla essential oil	Anthocyanidin/chitosan	Pickering nano-emulsion	Antioxidant activity, improved mechanical and barrier properties	[180]
Bay and rosemary essential oils	Zein	Electrospinning	Antibacterial activity against *S. aureus* and *L. monocytogenes*	[181]

**Table 5:** Key challenges and future directions in food nanotechnology.

Domains	Challenges	Future Directions	Ref.
Safety and toxicological	Metallic NPs (ZnO, TiO_2_) exhibit excellent antimicrobial properties; however, their migration into food can cause cytotoxic or genotoxic effects.	Safe concentration limits and long-term health impacts.	[208]
Regulatory and standardization	The lack of standardized regulations and testing for nanomaterials in food packaging is a significant challenge, recognized by both governments and academic institutions.	Comprehensive risk assessment frameworks, toxicological data, clear labeling, and updated legislation.	[209]
Controlled release and stability	Ensuring the stability and controlled release of nanoencapsulated ingredients (vitamins, antioxidants, and bioactive compounds) without compromising food quality.	Selecting suitable materials for controlled release, food quality, stability during processing, storage, and digestion.	[210]
Environmental and sustainability	Biodegradable packaging from natural polymers is eco-friendly but limited by its weak properties, including poor mechanical properties, and water sensitivity, and may also hinder recycling and biodegradation.	Eco-safe, environmentally compatible nanomaterials.	[211]
High cost and scalability	Carbon-based nanomaterials offer promise for smart packaging; however, high costs and technical challenges limit their large-scale application.	Cost-effective synthesis, material optimization, and scalable manufacturing processes.	[187]
Consumer acceptance and awareness	Concerns over safety and labeling transparency influence consumer acceptance of nanotechnology in food.	Education, transparent labeling, and robust safety evidence are necessary to build trust.	[212]

## Data Availability

Not applicable.
